# Prevalence, awareness, treatment, and control of hypertension and their risk factors in Shaanxi Province in 2004–18

**DOI:** 10.1038/s41598-023-28407-4

**Published:** 2023-02-13

**Authors:** Weihua Wang, Rina Sa, Shaonong Dang, Lin Qiu, Feng Liu

**Affiliations:** 1 Shaanxi Provincial Center for Disease Control and Prevention, No.3, Jiandong Road, Xi’an, Shaanxi People’s Republic of China; 2 School of Public Health, Xi’an Jiaotong University Health Science Center, Xi’an, People’s Republic of China

**Keywords:** Hypertension, Health care, Risk factors

## Abstract

To investigate trends in the prevalence, awareness, treatment and control of hypertension and their demographic determinants in Shaanxi Province. Six successive cross-sectional surveys on non-communicable chronic diseases and their risk factors were conducted between 2004 and 2018 in Shaanxi. Complex multistage stratified sampling was adopted to select participants. The information was collected through face-to-face interviews and on-site health examinations. Changes in hypertension prevalence and its management across survey years were estimated. Demographics associated with hypertension prevalence and its management was explored by multivariable logistic regression using pooled data from 2004 to 2018. The prevalence of hypertension increased from 16.71% in 2004 to 31.96% in 2018 with an estimated increase of 1.09% (95% CI 0.31–1.87) per year. However, the rate of awareness, treatment and control among these with hypertension was unexpectedly low and there were no significant change from 2004 to 2018. The corresponding changes were − 0.08% (95% CI − 0.85–0.69) per year for awareness, − 0.06% (95% CI − 1.11–1.00) per year for treatment, and − 0.23% (95% CI − 0.53–0.07) per year for control, respectively. Sensitivity analysis showed the same trend. Adults who were old, male, divorced/Widowed/Separated, retired were more likely to develop hypertension. Among these with hypertension, those who were more educated and retired were more likely to manage their hypertension compared with their counterparts. The overall hypertension prevalence from 2004 to 2018 increased rapidly, while awareness, treatment and control of hypertension remained unexpectedly low. This suggested urgent intervention should be implemented to improve hypertension control in Shaanxi Province.

## Introduction

Hypertension is the leading risk factor for varieties of diseases, such as cardiovascular diseases, stroke, and kidney disease^1–3^. Studies showed that high hypertension prevalence, relatively low hypertension treatment and control rates in China are common nationwide^4–7^. Control of hypertension has been a national public health priority in China^8,9^. Due to the largest developing country with large territory, the prevalence of hypertension and its management in China still highly varied across spatial scales^10^. Some studies showed the prevalence of hypertension increased slightly and the treatment remains at low levels within the decades in south and east of China, which are relatively economically developed areas^11,12^. However, data on hypertension from northwest of China, which was less developed, is scarce, and change of prevalence and its management of hypertension in the areas in recent years are uncertain.

Shaanxi is located in northwest of China with a population of 38 million. Its per capita disposable income was 17,395 yuan in 2015 below the national average 21,966.2 yuan, and far less than some developed areas in east of China 35,537 Yuan^13–16^. Shaanxi province therefore provides an opportunity to investigate the changing trend of hypertension prevalence and its management from less industrialized and urbanized areas in northwest China. We performed an analysis of 6 cross-sectional surveys on non-communicable chronic diseases conducted in Shaanxi between 2004 and 2018. Results on changing trends in hypertension and their risk factors in this area may inform central and local government on the hypertension prevention and control programs for Shaanxi Province, as well as for other developing areas.

## Results

### Characteristics of participants

From 2004 to 2018, the proportion of participants at 60 years old and above showed an upward trend (Z = 14.57, P < 0.01). The proportion of males is lower than that of females in all six surveys. The proportion of these educated ≥ 9 years ranged from 1.44% and 26.91% across the survey. The proportion of these married/cohabitants and employed was the highest in all six surveys. Table 1.

**Table 1:** Baseline of survey in Shaanxi Province from 2004 to 2018.

N (%)	2004	2007	2010	2013	2015	2018
Overall	1255 (100.0)	1542 (100.0)	3000 (100.0)	5969 (100.0)	6330 (100.0)	6213 (100.0)
Sex						
Male	540 (43.03)	752 (48.77)	1371 (45.70)	2746 (46.00)	3083 (48.70)	2787 (44.86)
Female	715 (56.97)	790 (51.23)	1629 (54.30)	3223 (54.00)	3247 (51.3)	3426 (55.14)
Age group						
18–44	717 (57.13)	774 (50.19)	1134 (37.80)	2364 (39.60)	1897 (29.98)	1448 (23.36)
45–59	376 (29.96)	516 (33.46)	1182 (39.40)	2246 (37.63)	2568 (40.59)	2536 (40.91)
≥ 60	162 (12.91)	252 (16.34)	684 (22.80)	1359 (22.77)	1862 (29.43)	2215 (35.73)
Education level (year)						
≤ 6	547 (43.62)	558 (36.19)	1201 (40.06)	2158 (36.22)	2959 (46.75)	3062 (49.28)
7–8	689 (54.94)	662 (42.93)	1000 (33.36)	2197 (36.87)	2234 (35.29)	2119 (34.11)
≥ 9	18 (1.44)	322 (20.88)	797 (26.58)	1603 (26.91)	1137 (17.96)	1032 (16.61)
Marital status						
Single	101 (8.05)	152 (9.86)	154 (5.15)	399 (6.69)	273 (4.31)	199 (3.2)
Married/co-habitat	1053 (83.97)	1244 (80.67)	2552 (85.29)	5071 (85.07)	5761 (91.01)	5582 (89.84)
Divorce/widow/separation	100 (7.97)	146 (9.47)	286 (9.56)	491 (8.24)	296 (4.68)	432 (6.95)
Occupation						
Employed	979 (78.07)	1299 (84.24)	2182 (72.78)	4244 (71.20)	4835 (76.38)	4564 (73.46)
Unemployed	201 (16.03)	133 (8.63)	603 (20.11)	1333 (22.36)	1170 (18.48)	1349 (21.71)
Retired	74 (5.90)	110 (7.13)	213 (7.10)	384 (6.44)	325 (5.13)	300 (4.83)
Residents						
Urban	420 (33.47)	311 (20.17)	600 (20.00)	2985 (50.01)	2711 (42.83)	3035 (48.85)
Rural	835 (66.53)	1231 (79.83)	2400 (80.00)	2984 (49.99)	3619 (57.17)	3178 (51.15)
BMI (kg/m^2^)	22.16 ± 3.16	22.87 ± 5.55	23.42 ± 3.55	24.11 ± 3.56	24.29 ± 3.47	24.36 ± 3.41
Cigarettes smoking						
Never	790 (63.25)	1017 (65.95)	2116 (70.53)	4092 (68.55)	4171 (65.89)	4283 (68.94)
Current	459 (36.75)	525 (34.05)	884 (29.47)	1877 (31.45)	2159 (34.11)	1930 (31.06)
Drinking						
Never	739 (59.26)	1057 (68.55)	2156 (71.87)	4239 (71.02)	4408 (69.64)	4566 (73.49)
Ever	508 (40.74)	485 (31.45)	844 (28.13)	1730 (28.98)	1922 (30.36)	1647 (26.51)
Mean blood pressure (mmHg)						
Systolic	120.78 ± 17.42	129.22 ± 20.01	135.42 ± 22.94	129.45 ± 21.25	136.41 ± 21.96	136.18 ± 20.93
Diastolic	74.24 ± 11.60	79.32 ± 10.6	81.39 ± 12.04	76.81 ± 11.58	79.23 ± 11.68	79.01 ± 11.42

### Prevalence of hypertension

The prevalence of hypertension increased from 16.71% in 2004 to 31.96% in 2018, with an estimated increase of 1.09% per year (95% CI 0.31–1.87). The increase of hypertension prevalence among males was 1.38% per year (95% CI 0.59–2.18), while no such trend found among females (0.79% per year, 95% CI − 0.03–1.62). The estimated increase per year was highest among these ≥ 60 years old (1.58% per year, 95% CI 0.22–2.93), educated ≤ 6 years (1.49% per year, 95% CI 0.57–2.40), as well as those divorced, widowed or separated (2.10% per year, 95% CI 0.65–3.55). The estimated increase per year was 1.77% (95% CI 1.10–2.44) in rural residents, and no such trend found among their urban counterparts (0.41% per year, 95% CI − 0.27–1.09). Table 2.

**Table 2:** Prevalence of hypertension in Shaanxi Province from 2004 to 2018.

% (95% CI)	2004	2007	2010	2013	2015	2018	Estimated prevalence change per year
Overall	16.71 (14.57–18.84)	19.21 (16.83–21.60)	38.01 (36.17–39.85)	32.69 (31.02–34.36)	32.08 (29.81–34.36)	31.96 (29.12–34.8)	1.09 (0.31–1.87)
Sex							
Male	16.16 (12.91–19.41)	18.14 (14.79–21.48)	34.73 (32.21–37.26)	33.83 (32.05–35.61)	31.88 (30.21–33.54)	35.82 (30.86–40.78)	1.38 (0.59–2.18)
Female	17.22 (14.42–20.02)	20.62 (17.28–23.95)	41.17 (38.78–43.56)	31.77 (30.15–33.38)	32.3 (30.67–33.92)	27.93 (25.52–30.34)	0.79 (− 0.03–1.62)
Age group							
18–44	7.67 (5.71–9.62)	11.95 (9.15–14.75)	19.11 (16.81–21.40)	14.08 (12.67–15.50)	16.02 (14.33–17.70)	18.51 (13.68–23.34)	0.69 (0.33–1.06)
45–59	27.80 (22.96–32.63)	33.43 (28.78–38.09)	45.67 (42.82–48.51)	37.13 (35.12–39.14)	44.31 (42.37–46.24)	42.28 (39.63–44.93)	1.00 (0–2.00)
≥ 60	40.76 (33.03–48.49)	49.81 (42.66–56.97)	64.22 (60.61–67.82)	58.47 (55.84–61.11)	69.93 (67.83–72.03)	63.05 (60.41–65.68)	1.58 (0.22–2.93)
Education level (year)							
≤ 6	22.35 (18.69–26.02)	25.84 (21.56–30.13)	46.93 (44.10–49.76)	41.66 (39.57–43.75)	45.09 (43.28–46.90)	41.51 (38.65–44.37)	1.49 (0.57–2.4)
7–8	12.40 (9.86–14.95)	15.60 (12.29–18.92)	30.24 (27.39–33.09)	29.20 (27.29–31.11)	27.50 (25.62–29.37)	31.85 (27.91–35.80)	1.4 (0.61–2.18)
≥ 9	27.65 (7.31–47.98 )	19.24 (13.69–24.78)	34.95 (31.63–38.26)	28.04 (25.83–30.26)	19.70 (17.36–22.05)	22.33 (15.01–29.64)	− 0.58 (− 1.82–0.66)
Marital status							
Single	1.58 (− 0.27–3.43)	8.58 (3.20–13.97)	25.47 (18.51–32.43)	12.85 (9.54–16.15)	14.4 (10.08–18.72)	14.08 (− 0.12–28.27)	0.81 (− 0.44–2.06)
Married/co-habitat	18.63 (16.15–21.11)	21.77 (19.06–24.49)	36.86 (34.99–38.73)	32.8 (31.50–34.10)	33.22 (31.99–34.45)	34.83 (32.53–37.14)	1.12 (0.46–1.78)
Divorce/widow/separation	26.33 (17.47–35.19)	24.52 (16.36–32.69)	55.88 (50.15–61.61)	53.13 (48.69–57.57)	67.42 (61.97–72.86)	47.12 (39.22–55.02)	2.1 (0.65–3.55)
Occupation							
Employed	13.85 (11.61–16.08)	18.73 (16.16–21.30)	35.41 (33.40–37.42)	30.92 (29.53–32.32)	31.25 (29.93–32.57)	30.24 (27.73–32.76)	1.18 (0.48–1.87)
Unemployed	17.72 (12.45–22.99)	11.59 (6.76–16.43)	40.88 (36.94–44.81)	28.04 (25.61–30.47)	30.98 (28.28–33.68)	33.94 (24.84–43.03)	1.06 (0.14–1.99)
Retired	49.24 (37.64–60.85)	53.33 (42.42–64.23)	65.42 (58.98–71.86)	56.03 (51.03–61.04)	60.00 (54.61–65.39)	59.84 (52.8–66.88)	0.72 (0.30–1.14)
Residents							
Urban	24.76 (20.42–29.09)	29.38 (23.86–34.91)	40.5 (36.56–44.44)	29.73 (28.08–31.38)	27.35 (25.65–29.04)	31.94 (28.16–35.71)	0.41 (− 0.27–1.09)
Rural	11.55 (9.41–13.70)	18.29 (15.75–20.84)	37.7 (35.76–39.64)	34.69 (32.97–36.41)	36.15 (34.56–37.73)	32.01 (28.94–35.07)	1.77 (1.10–2.44)
BMI (kg/m^2^)							
< 24	10.79 (8.82–12.77)	14.95 (12.44–17.46)	29.88 (27.71–32.06)	22.18 (20.1–24.27)	24.55 (21.7–27.4)	21.7 (18.91–24.49)	0.85 (0.19–1.51)
24–28	35.59 (29.16–42.01)	27.66 (22.06–33.26)	48.51 (44.91–52.11)	38.29 (35.39–41.18)	34.87 (31.33–38.42)	37.1 (33.38–40.82)	0.02 (− 0.72–0.77)
≥ 28	37.01 (22.06–51.97)	60.99 (46.61–75.38)	62.31 (56.3–68.32)	53.46 (48.79–58.13)	55.08 (47.26–62.9)	57.57 (47.24–67.91)	0.94 (− 0.5–2.39)
Cigarettes smoking							
Never	15.39 (12.86–17.92)	19.64 (16.66–22.62)	40.91 (38.67–43.15)	31.44 (29.44–33.44)	29.42 (26.83–32.01)	28.78 (26.36–31.19)	0.86 (0.06–1.65)
Current	18.97 (15.16–22.78)	18.5 (14.54–22.46)	31.5 (28.32–34.68)	35.63 (32.62–38.64)	37.54 (33.28–41.79)	38.63 (31.84–45.43)	1.56 (0.65–2.47)
Drinking							
Never	16.94 (14.14–19.74)	19.51 (16.64–22.39)	39.94 (37.74–42.13)	33.62 (31.69–35.56)	32.88 (29.97–35.79)	31.91 (28.37–35.44)	1.02 (− 0.06–2.09)
Ever	16.52 (13.2–19.85)	18.7 (14.51–22.88)	33.51 (30.17–36.85)	30.64 (27.41–33.88)	30.56 (26.95–34.18)	32.08 (27.44–36.72)	1.16 (0.64–1.68)
Standardized prevalence	15.72	21.14	32.00	26.44	32.35	32.15	

### Awareness, treatment, control of hypertension

The rate of awareness among participants with hypertension in Shaanxi Province ranged from 24.05% in 2007 to 43.82% in 2018, with no change per year from 2004 to 2018 (− 0.08% per year, 95% CI − 0.85–0.69). The awareness rate of hypertension for males and females was 25.27 and 21.49% in 2004, 20.07 and 26.09% in 2018, respectively. The hypertension awareness rate in urban and rural areas was 19.83 and 25.36% in 2004, 24.11 and 19.66% in 2018, respectively.

The rate of treatment among participants with hypertension was 21.69% in 2007 and 24.97% (95% CI 21.78–28.17) in 2018. The overall rate remained no change throughout years (− 0.06% per year, 95% CI − 1.11–1.00). The treatment rate for hypertension for males and females was 12.60 and18.46% in 2007, 15.90 and 20.79% in 2018, respectively. The treatment rate of hypertension in urban and rural was 22.91 and14.57% in 2007, 20.21 and 13.16% in 2018, respectively.

**Figure 1:**
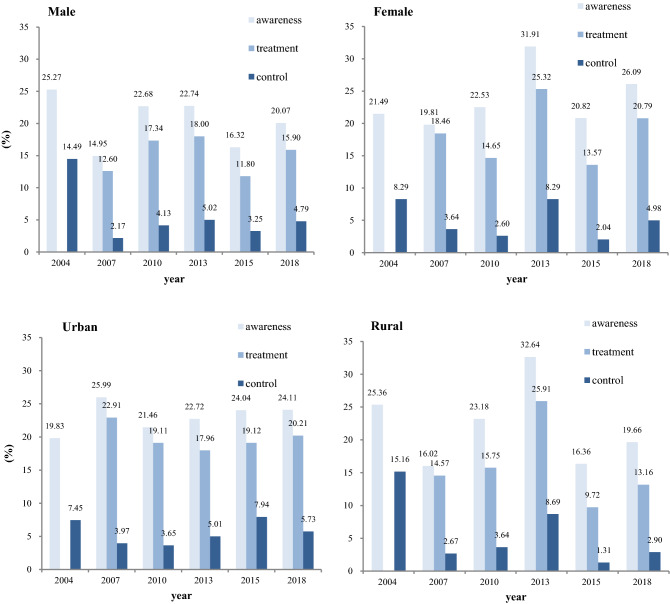
Awareness, treatment, control of hypertension in Shaanxi Province from 2004 to 2018 (data of treatment were not collected in 2004).

The rate of control among hypertensive adults was 11.58% in 2004 and 6.55% (95% CI 5.15–7.96) in 2018 with no change throughout years (− 0.23% (95% CI − 0.53–0.07)). The control rate for males and females was 14.49 and 8.29% in 2004, 4.79 and 4.98% in 2018, respectively. The control rate in urban and rural areas was 7.45 and 15.16% in 2004, 5.73 and 2.90% in 2018, respectively (Fig. 1).

### Associated factors

Logistic regression models identified demographic characteristics associated with hypertension prevalence, awareness, treatment and control. Participants who were older, male, with higher BMI, divorced/Widowed/Separated, retired were more likely to develop hypertension. While these with hypertension, more educated, retired, with lower BMI, were more likely to manage their hypertension compared with their counterparts. Table 3.

**Table 3:** Demographic characteristics associated with the prevalence, awareness, treatment and control of hypertension from 2004 to 2018.

New	Prevalence		Awareness		Treatment		Control	
	OR	95% CI	OR	95% CI	OR	95% CI	OR	95% CI
Sex								
Male	1.10	(0.92–1.31)	0.73	(0.55–0.98)	0.81	(0.59–1.11)	0.83	(0.58–1.17)
Age (year)								
≥ 60	1.00	(Ref)	1.00	(Ref)	1.00	(Ref)	1.00	(Ref)
45–59	0.13	(0.11–0.15)	0.34	(0.22–0.52)	0.26	(0.18–0.37)	0.83	(0.42–1.64)
18–44	0.41	(0.37–0.46)	0.87	(0.76–1.01)	0.78	(0.63–0.97)	1.12	(0.81–1.53)
Education (year)								
≥ 9	1.00	(Ref)	1.00	(Ref)	1.00	(Ref)	1.00	(ref)
7–8	1.35	(1.07–1.71)	0.71	(0.53–0.96)	0.73	(0.54–0.97)	0.64	(0.43–0.98)
≤ 6	1.09	(0.87–1.36)	0.80	(0.6–1.07)	0.85	(0.63–1.15)	0.71	(0.49–1.01)
Marital status								
Divorce/widow/se-paration	1.00	(Ref)	1.00	(Ref)	1.00	(Ref)	1.00	(Ref)
Single	0.58	(0.32–1.06)	0.29	(0.14–0.59)	0.29	(0.13–0.66)	0.32	(0.1–1.05)
Married/co-habitat	0.87	(0.74–1.03)	0.85	(0.69–1.04)	0.75	(0.63–0.88)	0.95	(0.61–1.49)
Occupation								
Retired	1.00	(Ref)	1.00	(Ref)	1.00	(Ref)	1.00	(Ref)
Employed	0.73	(0.61–0.87)	0.56	(0.36–0.87)	0.45	(0.38–0.53)	0.42	(0.29–0.6)
Unemployed	0.85	(0.68–1.06)	0.52	(0.32–0.85)	0.40	(0.31–0.52)	0.40	(0.27–0.6)
Residency								
Urban	0.89	(0.73–1.09)	1.02	(0.79–1.31)	1.23	(0.91–1.66)	1.19	(0.74–1.91)
BMI (kg/m^2^)								
≥ 28	1.00	(Ref)	1.00	(Ref)	1.00	(Ref)	1.00	(Ref)
24–28	0.43	(0.33–0.55)	0.91	(0.76–1.08)	0.97	(0.81–1.18)	1.26	(0.98–1.61)
< 24	0.19	(0.15–0.25)	0.58	(0.49–0.69)	0.56	(0.45–0.69)	1.13	(0.77–1.67)
Cigarettes smoking								
Never	0.85	(0.69–1.04)	0.97	(0.74–1.28)	1.03	(0.76–1.40)	0.85	(0.61–1.18)
Drinking								
Never	1.08	(0.86–1.35)	1.16	(0.86–1.56)	1.25	(0.85–1.83)	0.91	(0.61–1.35)

### Sensitivity analysis

**Figure 2:**
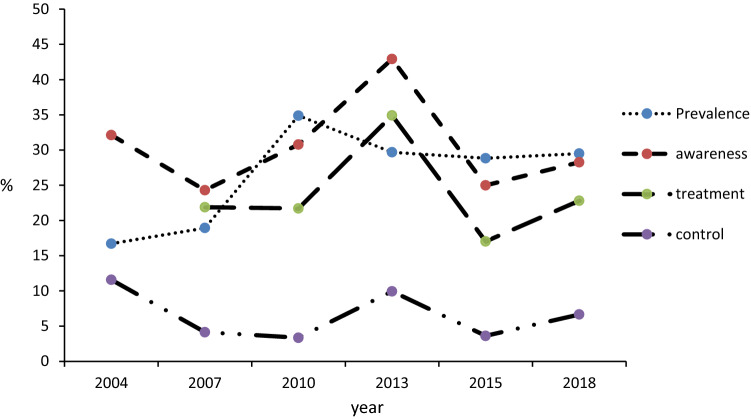
prevalence of hypertension, hypertension awareness, treatment and control in Shaanxi Province from 2004 to 2018. (Participants aged > 69 years old were excluded and data from 17 monitoring points in survey 2013 added to the sensitivity analysis).

To keep the age range of participants from the six surveys the same, we excluded these aged > 69 year old in survey of 2010, 2013, 2015 and 2018 and we also added observations from 7 provincial monitoring points in survey of 2013. The prevalence of hypertension increased from 2004 to 2018 (Z = 16.27, P < 0.01). However, the hypertension awareness (Z = 0.67, P = 0.51), treatment (Z = 1.51, P = 0.13) and control (Z = 1.59, P = 0.11) showed no any change. The results were generally similar to the main analysis (Fig. 2).

## Discussion

To our knowledge, few studies based on the multi-stage population-based survey demonstrate temporal distribution of hypertension prevalence and management in Shaanxi Province, Northwest China. We found the prevalence of hypertension increased rapidly over the past decade, while the awareness, treatment and control among hypertensive adults remained stable and unexpectedly low. There would be substantial hypertension burden in the coming years in this area if comprehensive public health intervention did not implement promptly.

From 2004 to 2018, we found the prevalence of hypertension in Shaanxi Province nearly doubled, with an increase of 1.09% per year. In contrast, the prevalence had decreased in developed countries, such as Finland and Germany in the past decade^17,18^. In the United States, the percentage of hypertensive adults with stage I or stage II hypertension decreased from 2003 to 2012^19^. Studies have shown in Asia, the prevalence of hypertension was significantly decreased in Korea from 1998 to 2014 and in Japan from 1975 to 2010^20,21^. Our results are consistent with both meta-analysis and survey studies that revealed increased hypertension prevalence in China over the last few decades^11,22,23^. However, the magnitude of increase in Shaanxi Province is much higher than the national average, which the prevalence of hypertension had increased from 18.0% in 2002 to 27.8% in 2013^24^.

The rate of hypertension awareness, treatment and control in our study was low and stable over the past decade; couple with the increased prevalence of hypertension. Although Chinese government has been taking several actions to control the prevalent hypertension, i.e. involving hypertension management in National basic public health service since 2009 and enhancing the investment per year, setting a goal for 60% of those treated get controlled by 2015. The present study suggested that the intervention might not as effective as expected, at least in Shaanxi Province. The awareness rate was 20% on average in these six surveys; lower than the national level 31.9% and eastern China, nonetheless the developed countries^12,25–28^. The treatment was below 20% and controls below 5% among those with hypertension, both of them was lower than the average level in developing countries^28,29^. Due to its less developed economy and relatively low investment to health care, lack of primary health care personnel and antihypertensive medication basically attributed to the low treatment and control rate. In addition, unhealthy lifestyles and underuse of antihypertensive drugs among hypertensive people may also contributed to the poor hypertension control^25,30^. Our analysis revealed that hypertension awareness, treatment and control in less developed areas in China were unexpectedly low and remained unchanged in the past decade. Thus policy makers ought to inform the geographic disparity of hypertension awareness and control. On one hand China should enhance expenditure on hypertension screening and public awareness programs, on the other hand government ought to target regions with high prevalence and low control such as Shaanxi Province, northwest China by investing in hypertension prevention and management programs, such as promoting structured physician education and improve guideline adherence.

There are many factors are associated with prevalence of hypertension and its management as we explored in the logistic regression. In the study, we found that the prevalence of hypertension rose with age in all surveys from 2004 to 2018. Concurrently, statistic demonstrated the population in this area was aging, with the percentage of population aged 65 years old or above increased from 7.65% in 2004 to 10.11% in 2015. This might have contributed to the prevalent hypertension and its consequences, such as cognitive impairment which the only hypertension-mediated organ damage in over 30% of hypertensive patients^31^. In the past decade, there was more than 20% population from rural areas to urban areas and the income over tripled due to urbanization and economic development^14,32^. Boom in economics and urbanization are related to lifestyle change, residents were more likely to have less physical activity and consumed more energy-dense diets, both of which contributed to overweight or obesity. We found that the increased overweight and obesity prevalence in the same period in both urban and rural areas possibly intensify the BP increase and hinder its control. Since the unchanged awareness, treatment among the hypertensive, the lifestyle changes may have been a major contributor to rapid increase of hypertension prevalence. We also found these who were more educated, retired, with lower BMI, with a relatively higher socio-economic status (SES) were more likely to have a better hypertension management. These findings were in line with other studies^33^.

This study has several limitations. One limitation is that we adopted KISH table to select participants in each household in survey 2004, 2007, 2010 and 2013, while all the family members in each household as participants were involved in 2015 and 2018. The sampling adjustment on representativeness and survey results needs to be evaluated further. The sample size enlarged in survey from 2013 to 2018, the unequal number of participants might have an impact on comparison between surveys. However, we calculated weight to represent the local population, in plus with standardizing all of the prevalence and rate, to eliminate the errors. Two readings were taken for survey 2004, whereas three readings were taken for others. We used the second reading for participants with two readings and the average of the last two readings for those with three readings in our analysis. The first reading is often higher than the second or third readings^29^. Research showed this inconsistency did not affect conclusions^23^. Inconsistency of sphygmomanometers over surveys is also a limitation. Standardized mercury sphygmomanometers were used in survey 2004, whereas Omron electronic sphygmomanometers were adopted in other surveys. This might have resulted in lower blood pressure levels and hypertension prevalence in 2004 than in other surveys. However, there is some strength in the study. The serial surveys were conducted in a large population resided in Shaanxi Province, a less developed areas in China. Moreover, the demographic characteristics of study population are relatively homogeneous. Risk factors on hypertension trend would be consistent over survey waves.

In conclusion, we found the overall hypertension prevalence from 2004 to 2018 increased rapidly in population in Shaanxi Province, northwest China. It is likely that the hypertension trend might not be stopped in a near future because of its accelerated urbanization and aging. The rate of hypertension awareness, treatment and control in this area remains at unexpectedly low levels and without any improvement over the 12-year period.

## Methods

The data that support the findings of this study are available from the corresponding author on reasonable request.

### Data and participants

The sample sizes of survey in 2010, 2013, 2015 and 2018 were calculated as following:

$$N\, = \,deff\,\frac{{u^{2} p\,\left( {1 - p} \right)}}{{d^{2} }}.$$

The confidence level (CI) is 95% (two-side), *u* = 1.96; the prevalence of diabetes 10.4% was selected as *P*, The design effect (deff) was 3, Relative error *r* = 20%, *d* = 20 × 10.4%. Considering the non-response rate is 15% in the survey.

For each survey, we conducted a complex multistage stratified sampling method to recruit participants. At the first sampling stage, two townships/streets were selected in each surveillance point, which is a district in a city or a county, by probability proportionate to size sampling (PPS) method. At the second stage, four administrative villages/communities were random selected in each township/street. One group was then randomly selected in each administrative village/community at the third stage. In each group, we randomly selected 40 households. KISH table was used to select participants in each household who had lived in their current residence for at least 6 months within a year in survey 2004, 2007, 2010 and 2013, whereas all the family members were selected from each household in 2015 and 2018. Survey in 2004 was conducted in two surveillance points in Shaanxi Province. We expanded the number of points in Shaanxi from 2 to 5 in 2007 and 2010, further to 10 in 2013, 2015 and 2018, respectively. Details of the establishment, design, sampling method and quality control had been reported elsewhere^34–38^. The surveillance was approved by the ethical review committee of the Chinese Center for Disease Control and Prevention and written informed consent was obtained from all participants at enrollment. The study was conducted in accordance with the Declaration of Helsinki.

### Data collection

A face-to-face interview was conducted by trained staff from local CDC (Centers for Disease Control and Prevention) and health care institutes using standardized questionnaires. Information of each participant on demographic characteristics, medical history and lifestyle factors was collected. Height, weight and blood pressure was measured on-site health examinations.

The blood pressure of each participant was measured at the non-dominant arm 3 times in succession with 1 min interval in a separate examination room after a 5-min rest, except 2 times were measured in 2004. We adopted an electronic upper arm blood pressure monitor (HBP-1300, Omron Healthcare, Inc., Kyoto, Japan) to measure it, except for a Standard mercury column mercury meter in 2004. The mean of the last two readings was used for analysis from 2007 to 2018. The second readings were used in 2004.

### Main variables

According to the *Chinese Guidelines for the Prevention and Treatment of Hypertension 2018*, hypertension is defined as systolic blood pressure (≥ 140 mmHg) and/or diastolic blood pressure (≥ 90 mmHg), or those with hypertension and takes anti-hypertension drugs in the past. The proportion of participants with hypertension in all the participants was defined as prevalence of hypertension. Participants who had been diagnosed with hypertension before the survey were deemed as awareness of hypertension. The rate of hypertension treatment is the proportion of participants with hypertension taking antihypertensive drugs in the past two weeks before the survey. The rate of hypertension control is the proportion of hypertensive patients whose systolic blood pressure < 140 mmHg and diastolic blood pressure < 90 mmHg.

Current smoking is defined as smokers used cigarettes every days or some day during the survey. Alcohol drinking is defined as consumed alcohol in the past 12 months. According to *the standard of Chinese guidelines for the prevention and control of overweight and obesity in adults*, BMI < 24 kg/m^2^ is considered normal or low weight, 24 kg/m^2^ ≤ BMI < 28 kg/m^2^ was considered overweight, and BMI ≥ 28 kg/m^2^ was considered obesity^39^.

### Quality control

In each survey, investigators had to be trained in both national and provincial level and those who were passed the test after training could conduct the survey. Anthropometric meters were standardized before the survey. A checking system including checking in the field by interviewers, checking by supervisors in the work group was adopted during the survey. Subjects were re-interviewed when logical questions and missing values were found.

### Statistical analysis

Since all the surveys were conducted by a complex multistage stratified sampling, we calculated weights of each survey, which was the product of sampling weights and post-stratified weights. We reported all the prevalence and confidence intervals (CI) with weights. Cochran-Armitage trend test was used to analyze the trend of year. We also estimated unadjusted changes in hypertension prevalence and its management rates across survey years. To explore the demographics associated with hypertension and its management in a robust way, we conducted multivariable logistic regression with stepwise approach using pooled data from 2004 to 2018. Prevalence, awareness, treatment and control were the outcome in the models. Sex (male/female), age (18–44/45–59/ ≥ 60), education level (≤ 6,7–8, ≥ 9 years), marital status (single/ (married/co-habitat)/ (divorce/widow/separation)), occupation (employed/unemployed/ retired), residency (urban/rural), BMI (< 24/24–28/ ≥ 28), cigarettes smoking (never/ever) and drinking (never/ever) were the co-variates in the model. Since there are differences in sex, age, education level and other characteristics throughout surveys, we also standardized the prevalence by using the 6th national population census in 2010.

The age range in 2004 and 2007 was 15–69 year old and the rest of surveys were all above 18 years. In order to verify the reliability of the results, we also did sensitivity analysis. The main prevalence and rates including participants at 18–69 years old at all five survey was recalculated in the study. SAS 9.4 was used for statistical analysis in our study. All *p* values were two sided and < 0.05 was considered statistically significant.

## Data Availability

The data that support the findings of this study are available from the corresponding author on reasonable request.
